# Fatal Visceral Leishmaniasis Caused by *Leishmania infantum*, Lebanon

**DOI:** 10.3201/eid2405.180019

**Published:** 2018-05

**Authors:** Rana El Hajj, Hiba El Hajj, Ibrahim Khalifeh

**Affiliations:** American University of Beirut Medical Center, Beirut, Lebanon

**Keywords:** visceral leishmaniasis, Leishmania infantum, parasites, cutaneous leishmaniasis, zoonoses, Middle East, Lebanon

## Abstract

Visceral leishmaniasis, a fatal disease if not treated, is caused by *Leishmania* parasites. This disease might be overlooked in the Middle East because of limited awareness and low incidence. We report 5 patients who died of visceral leishmaniasis in Lebanon and make recommendations to improve faster diagnosis and treatment.

Leishmaniasis is a parasitic disease characterized by different clinical manifestations depending on patient immune response and causative species (*1*). Visceral leishmaniasis, the most severe form, is fatal if untreated (*2*). This disease is caused by *Leishmania donovani*, which is endemic to Africa and Asia, causes anthroponotic visceral leishmaniasis, and is associated with high mortality rates (*3*). However, *L. infantum*, which undergoes zoonotic transmission, is associated with fewer deaths and is endemic to Latin America and the Middle East (*4*). This species shows a mortality rate of 6% for children (*5*).

In the Middle East, cutaneous leishmaniasis is the most common endemic form of leishmaniasis and is caused mainly by *L. tropica* and *L. major* (*6*). *L. infantum* is reported to cause cutaneous leishmaniasis and visceral leishmaniasis in Syria, but only cutaneous leishmaniasis in Lebanon (*7*,*8*). During 1958–2014, visceral leishmaniasis showed a low incidence in Syria; 17 cases were reported in 2008 and 36 cases in 2014 (*9*,*10*). However, no molecular or biochemical typing was performed to identify the causative species and strains (*11*). Moreover, visceral leishmaniasis caused by *L .infantum* has not been reported in Lebanon (*12*).

Recently, displacement of refugees during the ongoing crisis in Syria resulted in a massive population migration and spread of communicable diseases, including cutaneous leishmaniasis (*6*,*11*). In Lebanon, 2,420 families from Syria were given a diagnosis of cutaneous leishmaniasis. As of April 2017, a total of 2,057 (85%) of these families were infected with *L. tropica* and 363 (15%) with *L. major* (*6*). These infections indicate the need for early diagnosis of visceral leishmaniasis and prevention of deaths in the Middle East. We report visceral leishmaniasis in refugees from Syria in Lebanon who acquired *L. infantum* in Syria. 

## The Study

Five refugee children from Syria (age range 2–11 years) died of visceral leishmaniasis during 2014–2017 because of a late diagnosis and lack of awareness of this disease in Lebanon. All 5 children had migrated from the northern coast of Syria to Lebanon and had resided in Beirut for an average of 9 months (range 7–11 months). Visceral leishmaniasis developed 4–6 months after they left Syria. Further investigations showed that the siblings of 3 of these patients were infected while staying in Syria.

All patients visited medical institutions in Lebanon and had fever, abdominal distension, ascites, hepatosplenomegaly, and pancytopenia (Table). Three patients were given incorrect diagnoses of leukemia and were given steroids and blood transfusions. The remaining 2 patients were given incorrect diagnoses of a hemophagocytic syndrome with an idiopathic etiology and treated accordingly.

**Table:** Clinical characteristics of 5 patients with visceral leishmaniasis, Lebanon, 2014−2017*

Patient no.	Age, y/sex	Abdominal distension	Hepatosplenomegaly	Fever	Ascites	Blood	Lung infiltrates	Insect bite	Delay in diagnosis, mo
1	11/F	+	+	+	+	Pancytopenia	+	+	4
2	7/M	+	+	+	+	Pancytopenia	+	−	5
2	5/M	+	+	+	+	Pancytopenia	+	−	6
4	4/M	+	+	+	+	Pancytopenia	+	−	5
5	2/ F	+	+	+	+	Pancytopenia	+	−	4

*−, negative; +, positive.

These 5 patients were then reevaluated at the American University of Beirut Medical Center (Beirut, Lebanon) after an average of 4.8 months. Microscopic examination of bone marrow aspirates and smear specimens showed a few scattered amastigotes within macrophages for 2 patients (Figure). 

**Figure:**
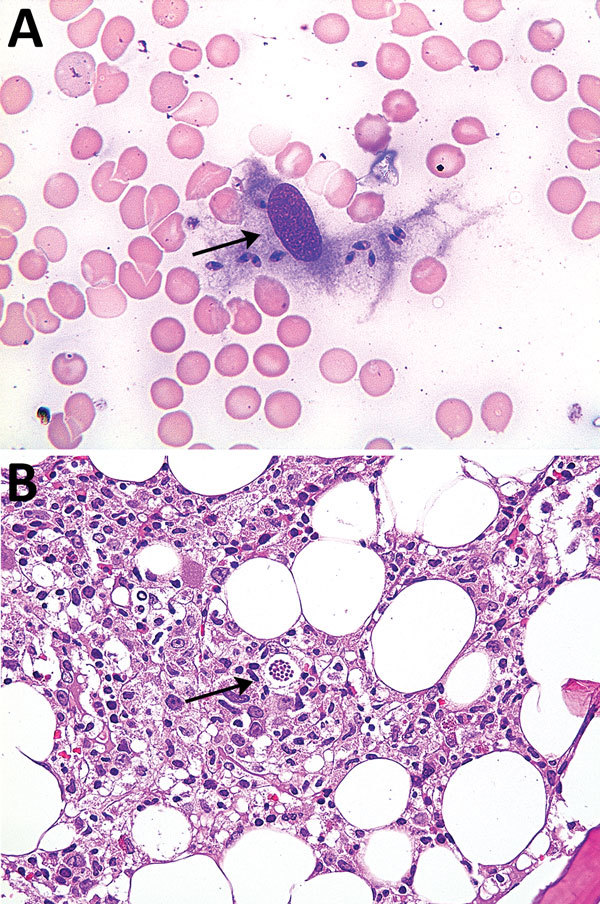
Bone marrow smear sample (A) and bone marrow aspirate (B) for patient 2 with visceral leishmaniasis caused by *Leishmania infantum*, Lebanon. Arrows show amastigotes within macrophages. Panel A, Wright Giemsa stain, original magnification x400; panel B, hematoxylin and eosin stain, original magnification x200.

A diagnosis of visceral leishmaniasis caused by *L. infantum* was confirmed by PCR amplification of the internal transcribed spacer 1 region of the parasite (*13*), followed by restriction fragment length polymorphism analysis of the internal transcribed spacer 1 region amplicon (*14*). This analysis specifically distinguishes *L. infantum* from other *Leishmania* species. After confirmation of visceral leishmaniasis, the patients were treated with Abelcet (amphotericin B lipid complex) (Teva Pharma BV, Harlow, UK) according to the manufacturer’s guidelines. However, the delay in diagnosis led to an advance disease stage and lack of response to treatment, followed by death.

## Conclusions

We report 5 children among refugees from Syria in Lebanon who died of visceral leishmaniasis caused by *L. infantum*. Our report provides insightful baseline information about knowledge, practices, and control regarding this disease. The combination of hepatosplenomegaly, fever, and pancytopenia should raise the suspicion for visceral leishmaniasis and result in prompt intervention. These interventions might prevent misdiagnosis and enable appropriate treatment strategies at early stages of the disease to reduce the number of deaths.
